# Do clinical guidelines facilitate or impede drivers of treatment in Fabry disease?

**DOI:** 10.1186/s13023-022-02181-4

**Published:** 2022-02-08

**Authors:** Derralynn A. Hughes, Patrício Aguiar, Olivier Lidove, Kathleen Nicholls, Albina Nowak, Mark Thomas, Roser Torra, Bojan Vujkovac, Michael L. West, Sandro Feriozzi

**Affiliations:** 1 Lysosomal Storage Disorders Unit, Institute of Immunity and Transplantation, Royal Free Hospital, Royal Free London NHS Foundation Trust, Rowland Hill Street, London, NW3 2PF UK; 2 Department of Haematology, University College London, London, UK; 3 Inborn Errors of Metabolism Reference Center, North Lisbon Hospital Center, Lisbon, Portugal; 4 Medicine Department, Faculty of Medicine, Lisbon University, Lisbon, Portugal; 5 Department of Internal Medicine-Rheumatology, Croix Saint Simon Hospital, Paris, France; 6 Department of Nephrology, Royal Melbourne Hospital, Parkville, VIC Australia; 7 Department of Medicine, University of Melbourne - Parkville Campus, Parkville, VIC Australia; 8 Department of Endocrinology and Clinical Nutrition, University Hospital Zurich and University of Zurich, Zurich, Switzerland; 9 Department of Internal Medicine, Psychiatry University Hospital Zurich, Zurich, Switzerland; 10 Department of Nephrology, Royal Perth Hospital, Perth, WA Australia; 11 Inherited Renal Diseases Unit, Fundacio Puigvert, University Autónoma de Barcelona, Barcelona, Spain; 12 Department of Internal Medicine, General Hospital Slovenj Gradec, Slovenj Gradec, Slovenia; 13 Department of Medicine, Dalhousie University, Halifax, NS Canada; 14 Division of Nephrology, Belcolle Hospital, Viterbo, Italy

**Keywords:** Fabry disease, Guideline, Consensus, Renal, Cardiac, Neurological, Patient-reported outcome, Treatment initiation, Enzyme replacement therapy, Chaperone therapy

## Abstract

**Background:**

Variable disease progression confounds accurate prognosis in Fabry disease. Evidence supports the long-term benefit of early intervention with disease-specific therapy, but current guidelines recommend treatment initiation based on signs that may present too late to avoid irreversible organ damage. Findings from the ‘PRoposing Early Disease Indicators for Clinical Tracking in Fabry Disease’ (PREDICT-FD) initiative included expert consensus on 27 early indicators of disease progression in Fabry disease and on drivers of and barriers to treatment initiation in Fabry disease. Here, we compared the PREDICT-FD indicators with guidance from the European Fabry Working Group and various national guidelines to identify differences in signs supporting treatment initiation and how guidelines themselves might affect initiation. Finally, anonymized patient histories were reviewed by PREDICT-FD experts to determine whether PREDICT-FD indicators supported earlier treatment than existing guidance.

**Results:**

Current guidelines generally aligned with PREDICT-FD on indicators of renal involvement, but most lacked specificity regarding cardiac indicators. The prognostic significance of neurological indicators such as white matter lesions (excluded by PREDICT-FD) was questioned in some guidelines and excluded from most. Some PREDICT-FD patient-reported signs (e.g., febrile crises) did not feature elsewhere. Key drivers of treatment initiation in PREDICT-FD were: (A) male sex, young age, and clinical findings (e.g., severe pain, organ involvement), (B) improving clinical outcomes and preventing disease progression, and (C) a family history of Fabry disease (especially if outcomes were severe). All guidelines aligned with (A) and several advocated therapy for asymptomatic male patients. There was scant evidence of (B) in current guidance: for example, no countries mandated ancillary symptomatic therapy, and no guidance advocated familial screening with (C) when diagnosis was confirmed. Barriers were misdiagnosis and a lack of biomarkers to inform timing of treatment. Review of patient histories generally found equal or greater support for treatment initiation with PREDICT-FD indicators than with other guidelines and revealed that the same case and guideline criteria often yielded different treatment recommendations.

**Conclusions:**

Wider adoption of PREDICT-FD indicators at a national level could promote earlier treatment in Fabry disease. Clearer, more concise guidance is needed to harmonize treatment initiation in Fabry disease internationally.

**Supplementary Information:**

The online version contains supplementary material available at 10.1186/s13023-022-02181-4.

## Background

Fabry disease (FD) is an X-linked, inherited disorder that is estimated to affect up to 1 in 40,000 individuals [1]. Mutations in the *GLA* gene encoding α-galactosidase A (α-Gal A) can cause lysosomal α-Gal A deficiency and accumulation of globotriaosylceramide (Gb3) and globotriaosylsphingosine (LysoGb3) [2]. Nearly 1000 *GLA* variants have been identified, and the effects of several on α-Gal A enzyme activity characterized, but the significance of many remains unknown [2]. There is considerable variation among individuals with FD in both symptomatic presentation and rate of disease progression [1]. Features characteristic of hemizygous male patients in the early stages of classical disease include neuropathic pain, abnormal hidrosis, gastrointestinal dysfunction, angiokeratoma, cornea verticillata, and microalbuminuria; subsequently, progressive renal, cardiac, and neurological complications can occur. Presentation in classical heterozygous female patients is more variable and generally less severe than in male patients, and pathology in non-classical forms of FD in both sexes may be confined to one organ system [1, 3–5]. Judging prognosis and, thus, when to initiate FD-specific treatment is a clinical challenge complicated by evidence that the best outcomes are associated with early treatment initiation. Long-term studies of patients with FD who initiated disease-specific therapy at an early stage have shown that it has a stabilizing effect on renal and cardiac parameters [6, 7]. Studies in vitro have implicated LysoGb3 in the formation of fibrosis and, in particular, the presence of renal or cardiac fibrosis on biopsy seems to mark a pivotal stage in FD after which disease-specific therapy becomes less effective [8]. One study found that plasma LysoGb3 levels 1 year after disease-specific therapy initiation were lower in men with classical FD who started treatment before age 25 years than in those treated later in life [9]; another found elevated baseline levels of LysoGb3 were associated with subsequent increased risk of adverse outcomes in patients with FD [10]. However, it remains unclear whether treatment-related reductions in LysoGb3 levels are associated with improved long-term outcomes.

The PRoposing Early Disease Indicators for Clinical Tracking in Fabry Disease (PREDICT-FD) initiative established expert consensus on indicators of FD progression that clinicians can use to inform treatment-initiation decisions [11]. A key motivation was to investigate whether collective clinical experience supports initiation of FD-specific treatment based on signs and symptoms that may present at an earlier stage of disease than do those underpinning existing guidance [11]. There are two approved FD-specific treatment modalities: intravenous enzyme replacement therapy (ERT) and oral chaperone therapy. The ERT agalsidase alfa (Replagal®) is licensed in Australia and Europe [12, 13] for use in FD from age 6.5–7 years, and the ERT agalsidase beta (Fabrazyme®) is licensed in Australia, Europe and North America from age 8 years [14–16]. Chaperone therapy with migalastat (Galafold®) is only suitable for patients with an amenable α-Gal A mutation and is licensed for use in patients aged 16 years or older in Australia, 12 years or older in Europe, and in adults in North America [17–19]. Investigational treatments in FD include pegunigalsidase alfa, a less immunogenic version of agalsidase alfa with an extended elimination half-life. Substrate reduction therapies, using glucosylceramide synthase inhibitors that prevent Gb3 accumulation, are in late-stage clinical development, and both ex vivo and in vivo gene therapies are under investigation: ex vivo approaches are in early clinical development and in vivo gene therapy methods have yielded encouraging results in animal models [20].

**Fig. 1:**
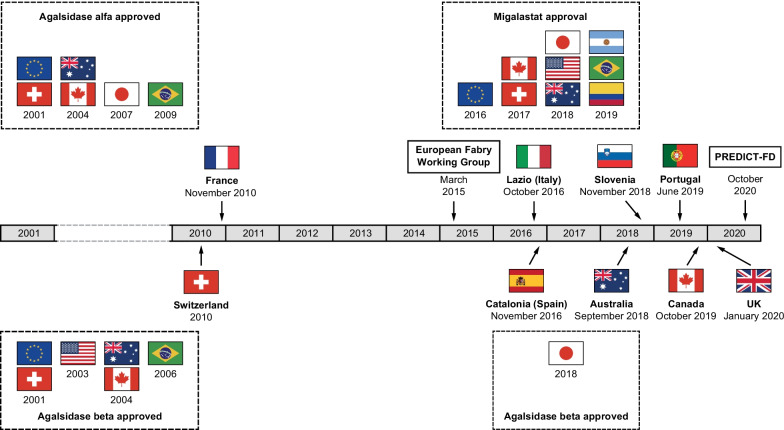
Timeline of publication of latest guidelines for FD and of disease-specific therapy approvals. As of December 31, 2017, agalsidase alfa was approved in 58 countries, excluding the USA. FD, Fabry disease

As well as examining the clinical case for appropriate FD-specific treatment initiation [11], PREDICT-FD required participants to suggest and to vote on factors that may drive or impede treatment initiation. Here, we report the consensus reached on these drivers of and barriers to treatment initiation and examine whether current treatment guidelines might contribute to delays in starting treatment. Accordingly, we compared the PREDICT-FD consensus with FD guidelines from different countries and with guidance issued by the European Fabry Working Group (EFWG) in 2015 [1]. The timelines of when these guidelines were issued and when disease-specific therapies were approved internationally are shown in Fig. 1; unpublished guidelines are summarized in Additional file 1: Table S1. Finally, as a preliminary examination of whether guideline variations may delay FD-specific treatment initiation, we consider some examples of treatment recommendations by a subset of panel members applying different guidelines to the same anonymized medical histories of patients with FD.

## Results

### PREDICT-FD consensus signs and symptoms and guidance for treatment initiation

The PREDICT-FD initiative achieved consensus on 27 early signs and symptoms of FD that might influence the initiation of FD-specific treatment at an earlier stage than is generally practiced [11]. Tables 1, 2, 3, and 4 summarize signs and symptoms identified in PREDICT-FD and those stipulated in different treatment guidelines. Treatment eligibility by organ involvement across the various guidelines is summarized in Additional file 1: Table S2.

**Table 1:** Renal signs: guideline stipulations and PREDICT-FD consensus [11]

	Histological damage (kidney biopsy)	Podocyte inclusions	Proteinuria	Elevated urine ACR (including microalbuminuria)	Abnormal GFR^a^	Decline in iohexol GFR	Renal dialysis or transplant
PREDICT-FD^b^ [11]	+	+	–	+	+	+	–
EFWG^c^ [1]	No consensus	No consensus	M, I; F, IIB	M, I; F, IIB	CKD stage 2: cM, I; ncM, IIA; cF, IIA; ncF, IIB CKD stage 3: cM, I; ncM, IIB; cF, IIB; ncF, IIB	–	Dialysis, do not withhold treatment Transplant, NA
Australia [24]	All^d^	–	M, > 150 mg/d F, > 300 mg/d	M^e,f^ > ULN	–	–	–
Canada^g^ [21]	M, F^h^	Renal pathology: M, major criterion F, minor criterion	All, ≥ 500 mg/d^i^ All, ≥ 300 mg/d^i^	–	All, CKD stage ≥ 2^j^	Decline in mGFR	All (considered CKD stage 5)
Catalonia (Spain)	–	–	All, > 300 mg/d	All	All, CKD stage ≥ 2^k^	–	All
France^l^ [23, 25]	F	–	F, > 1 g/d	–	F, moderate-to-severe	–	All
Portugal [22]	Confirmatory biopsy if needed, all asymptomatic	Confirmatory biopsy if needed, all asymptomatic	All	All	All, CKD stage ≥ 2	–	–
Slovenia (FCGHSG)	Confirmatory biopsy if needed in cF and in late-onset adults^m^	–	All	All	All CKD	–	–
Switzerland^n^	F^o^	F^n^	F, > 300 mg/d^p^	–	–	–	All^n^
UK^q^ [26]	–	–	All, > 300 mg/d^r^	–	All, CKD stage 2 and 3^s^	–	–

Unpublished guidelines are summarized in Additional file 1: Table S1

^a^CKD: stage 2, 60–90 mL/min/1.73 m^2^; stage 3, 45–60 mL/min/1.73 m^2^

^b^Consensus was reached that FD-specific treatment should be initiated at diagnosis in male patients aged 16 years or older who are asymptomatic for organ involvement, in boys younger than 16 years old with early indicators of organ involvement, and in all patients with guideline indicators of organ involvement

^c^Recommendations are based on class of evidence assigned: class I, treatment recommended or indicated; class IIA, treatment should be considered; class IIB, treatment may be considered; class III, treatment not recommended

^d^Including disease due to long-term renal accumulation of glycosphingolipids

^e^In two samples separated by at least 1 day

^f^In male patients, guideline abnormal albumin threshold of > 20 µg/minute is approximately equivalent to the threshold for microalbuminuria (> 30 mg/d)

^g^Treatment initiated based on one major or two minor criteria. Minor criteria not shown are renal tubular dysfunction and hypertension for at least 1 year

^h^If biopsy is indicated, glomerular sclerosis, tubulointerstitial atrophy, fibrosis, or vascular sclerosis constitute a major criterion in male patients and a minor criterion in female patients; biopsy not required for treatment initiation

^i^Major criterion: persistently ≥ 500 mg/d/1.73m^2^; minor criterion: ≥ 300 mg/d/1.73m^2^ in isolation or > normal and persisting for at least 1 year

^j^Major criterion: CKD stage 2 based on three consistent eGFRs over at least 4 months or stages 3–5 based on two consistent eGFRs over at least 2 months using CKD-EPI formula [27] in adults and the Counahan–Barrett formula [28] in children; also ≥ 15% decrease in GFR or slope greater than the age-related normal among those with hyperfiltration (GFR ≥ 135 mL/min/1.73 m^2^) determined by nuclear medicine techniques. Minor criterion: hyperfiltration in two measurements at least 1 month apart

^k^Reduced rate in at least three determinations by CKD-EPI equation [27]

^l^All male patients with a confirmed FD diagnosis should be offered ERT from age 18 years; ERT may be considered in children (6–18 years) with organic renal involvement

^m^Also if necessary in asymptomatic boys with a classical mutation

^n^ERT is practically always indicated in men, even those with mild symptoms and low organ involvement, and in patients undergoing hemodialysis or with a kidney transplant

^o^Relevant, histologically proven Gb3 deposits in kidney or heart biopsies

^p^Regardless of CKD stage

^q^FD-specific therapy should be considered in male patients with classical mutations at diagnosis; tabulated additional considerations apply to male and female patients with later-onset disease

^r^Persistent proteinuria > 300 mg/d in male patients; use anti-proteinuria medication for at least 12 months if proteinuria is the only presentation in female patients

^s^CKD stage 2 based on three or more consistent GFR estimates over at least 12 months and GFR slope greater than the age-related normal; CKD stage 3 based on two or more consistent GFR estimates over at least 6 months

+ , achieved consensus in PREDICT-FD; ACR, albumin–creatinine ratio; cF, female patient(s) with classical disease; cM, male patient(s) with classical disease; CKD, chronic kidney disease; CKD-EPI, CKD-Epidemiology Collaboration; d, day; EFWG, European Fabry Working Group; eGFR, estimated GFR; F, female patient(s); FCGHSG, Fabry Center, General Hospital Slovenj Gradec; FD, Fabry disease; Gb3, globotriaosylceramide; GFR, glomerular filtration rate; M, male patient(s); mGFR, measured GFR; ncF, female patient(s) with non-classical disease; ncM, male patient(s) with non-classical disease; NA, not available; ULN, upper limit of normal

**Table 2:** Cardiac signs: guideline stipulations and PREDICT-FD consensus [11]

	Early indicators of histological damage (heart biopsy)	Markers of early systolic/diastolic dysfunction	Elevated serum cardiac troponin	Early indicators of LVH	Late Gd+ on cMRI	Elevated serum NT-proBNP	Reduced myocardial T1 relaxation time on cMRI	Abnormal ECG	Abnormal echocardiogram	Abnormal wall motion on echocardiogram	Symptomatic cardiac disease
PREDICT-FD^a^ [11]	+ (NR)	+	+	+	+	+	+	+	+	+	–
EFWG^b^ [1]	–	–	–	Wall thickness > 12 mm with minimal/no fibrosis All, I	–	–	–	Rhythm disturbances All, I	–	–	–
Australia [24]	All	All^c^	–	All^d^	All^d^	–	All^c^	All^c^	All^d^	–	–
Canada^e^ [21]	Confirmatory diagnosis	Grade 2 or 3 diastolic dysfunction^f^	> 2 × ULN	Wall thickness: M, > 12 mm F, > 11 mm LVH Romhilt–﻿Estes score > 5^g^	Left ventricular wall	> ULN	1.5 T magnet M, < 901 ms F, 916 ms	Conduction/rhythm abnormal^h^	Diastolic filling abnormal Left atrium > 34 mL/m^2^ Moderate-to-severe mitral or aortic insufficiency Abnormal longitudinal strain gradient left ventricle		–
Catalonia (Spain)	–	All^i^	–	All^i,j^	All^k^	–	All^j^	All^j^	All^i^	All^i^	–
France^l^ [23, 25]	F^m^	–	–	–	–	F^m^	–	F^m^	F^m^	–	–
Portugal [22]	–		–	LVH in adults Cardiomyopathy in children	Myocardial fibrosis	–	–	All, arrhythmia Adults, conduction disturbance	–	–	Dyspnea, palpitations, syncope, thoracic pain
Slovenia (FCGHSG)	Confirmatory biopsy if needed in cF and in late-onset adults^n^	Diastolic dysfunction	–	Hypertrophic cardiomyopathy	Signs of fibrosis	–	–		Signs of fibrosis by speckle tracing		
Switzerland^o^	F^p^	F^q^	–	F^q^	–	–	F^q^	F^q^	–	–	–
UK^r^ [26]	–	–	–	Wall thickness: M, > 13 mm F, > 12 mm	All	–	–	–	All^s^	–	–

Unpublished guidelines are summarized in Additional file 1: Table S1

^a^Consensus was reached that FD-specific treatment should be initiated at diagnosis in male patients aged 16 years or older who are asymptomatic for organ involvement, in boys younger than 16 years old with early indicators of organ involvement, and in all patients with guideline indicators of organ involvement

^b^Recommendations are based on class of evidence assigned: class I, treatment recommended or indicated; class IIA, treatment should be considered; class IIB, treatment may be considered; class III, treatment not recommended

^c^Significant life-threatening arrhythmia or conduction defect

^d^LVH as evidenced by cMRI or echocardiogram data, in the absence of hypertension

^e^Treatment initiated based two criteria. Many cardiac manifestations may be attributable to hypertension, so this must be ruled out or treated for 12 months. One additional criterion not shown in the table: abnormal base–apex circumferential strain gradient on cMRI

^f^American Society of Echocardiography and/or the presence of speckle tracking abnormalities

^g^Additional criteria: LVM increase of 5 g/m^2^/y based on three measurements over at least 12 months; LVMI ≥ 20% above normal

^h^Atrioventricular block, short PR interval, left bundle branch block, ventricular or atrial tachyarrhythmias, sinus bradycardia in the absence of negative chronotropic drugs or other causes

^i^Echocardiographic changes: increased LVM, systolic or diastolic dysfunction, echocardiogram with persistently altered Doppler tissue

^j^Electrocardiographic changes; LVH; arrhythmia

^k^Alteration in cMRI suggestive of deposit

^l^All male patients with a confirmed FD diagnosis should be offered ERT from age 18 years; ERT may be considered in children (6–18 years) with cardiac involvement

^m^Treatment should be offered to women who develop cardiomyopathy; guideline does not specify how cardiomyopathy should be diagnosed, so various methods of diagnosis have been included except cMRI

^n^Also if necessary in asymptomatic boys with a classical mutation

^o^ERT is practically always indicated in men, even those with mild symptoms and low organ involvement, and in patients undergoing hemodialysis or with a kidney transplant

^p^Relevant, histologically proven Gb3 deposits in kidney or heart biopsies

^q^Manifest diastolic dysfunction, LVH, arrhythmias, attributable to cardiac involvement in FD

^r^FD-specific therapy should be considered in male patients with classical mutations at diagnosis; tabulated additional considerations apply to male and female patients with later-onset disease

^s^LVMI above normal for age and sex by 2D echocardiogram/cMRI

+ , achieved consensus in PREDICT-FD; 2D, two-dimensional; cF, female patient(s) with classical disease; cMRI, cardiac magnetic resonance imaging; ECG, electrocardiogram; EFWG, European Fabry Working Group; F, female patient(s); FCGHSG, Fabry Center, General Hospital Slovenj Gradec; FD, Fabry disease; Gb3, globotriaosylceramide; Gd+ , gadolinium enhancement; LVH, left ventricular hypertrophy; LVM, left ventricular mass; LVMI, LVM index; M, male patient(s); NR, achieved consensus but not recommended for safety reasons; NT-proBNP, N-terminal pro-natriuretic brain peptide; ULN, upper limit of normal; y, year

**Table 3:** Neurological signs: guideline stipulations and PREDICT-FD consensus [11]

	White matter lesions	Neuropathic pain	Painful GI symptoms suggestive of neuropathy	Stroke/TIA	Sudden onset unilateral hearing loss	Acute ischemic optic neuropathy	Silent cerebral infarct on MRI
PREDICT-FD^a^ [11]	–	+	+	+^b^	No consensus	–	–
EFWG^c^ [1]	All, IIB	All, IIA^d^	–	All, IIA	All, IIB^e^	–	–
Australia [24]	–	All^f^	–	All^g^	–	–	–
Canada^h^ [21]	–^i^	–^j^	–	All	All	All	–
Catalonia (Spain)	–	All^k^	–	All^l^	–	–	–
France^m^ [23, 25]	–	Children^n^		F, children	F,^o^ children^p^	–	–
Portugal [22]	All adults	All	Abdominal pain in children	All adults	All adults	–	All adults
Slovenia (FCGHSG)	All^q^	All	All^r^	All^q^	–	–	–
Switzerland^s^	–	F^t^	F	F^u^	–	–	–
UK^v^ [26]	–	All^w^	All^w^	–	–	–	–

Unpublished guidelines are summarized in Additional file 1: Table S1

^a^Consensus was reached that FD-specific treatment should be initiated at diagnosis in male patients aged 16 years or older who are asymptomatic for organ involvement, in boys younger than 16 years old with early indicators of organ involvement, and in all patients with guideline indicators of organ involvement

^b^Originally classified under “Other” [11]

^c^Recommendations are based on class of evidence assigned: class I, treatment recommended or indicated; class IIA, treatment should be considered; class IIB, treatment may be considered; class III, treatment not recommended

^d^If neuropathic pain is controlled and does not interfere with activities of daily living, all classified as IIB

^e^Age-adjusted hearing loss

^f^Uncontrolled chronic pain despite the use of maximum doses of appropriate analgesia and antiepileptic medications for peripheral neuropathy

^g^Ischemic vascular disease

^h^Treatment initiated based on one criterion

^i^Clinical significance of imaging abnormalities (white matter lesions, vessel dolichoectasia, cerebral microbleeds) alone is unclear and not an indication for ERT

^j^Pain in isolation does not warrant ERT. A 12-month trial of ERT may be considered based on agreed outcomes (e.g. reduced need for analgesics, reduced school/work time lost, reduced hospital admission for pain crises)

^k^Chronic pain, uncontrolled with drugs, that alters quality of life

^l^Ischemic cardiopathy; imaged vascular ischemic lesions attributable to FD

^m^All male patients with a confirmed FD diagnosis should be offered ERT from age 18 years

^n^Major painful crises refractory to analgesic treatment with carbamazepine, diphenylhydantoin, gabapentin, amitryptiline in children aged 6–18 years

^o^Severe cochlear damage

^p^Cochleo-vestibular involvement (hearing loss assessed by audiogram; vertigo of vestibular origin) in children aged 6–18 years

^q^Central and/or autonomic nervous system involvement consistent with FD

^r^Treatment initiation in classical male patients and girls with abdominal pain and postprandial diarrhea; additional confirmation of disease progression required in adult cF and in both sexes with the late-onset phenotype

^s^ERT is practically always indicated in men, even those with mild symptoms and low organ involvement, and in patients undergoing hemodialysis or after kidney transplantation

^t^Therapy-resistant pain

^u^Cerebrovascular manifestations (insult, transient ischemic attack); dizziness

^v^FD-specific therapy should be considered in male patients with classical mutations at diagnosis; tabulated additional considerations apply to male and female patients with later-onset disease

^w^Uncontrolled pain or GI symptoms requiring altered lifestyle or interfering with quality of life

+ , achieved consensus in PREDICT-FD; cF, female patient(s) with classical disease; EFWG, European Fabry Working Group; ERT, enzyme replacement therapy; F, female patient(s); FCGHSG, Fabry Center, General Hospital Slovenj Gradec; FD, Fabry disease; GI, gastrointestinal; MRI, magnetic resonance imaging; TIA, transient ischemic attack

**Table 4:** Patient-reported and other signs: guideline stipulations and PREDICT-FD consensus [11]

	Angiokeratoma	Organ biopsy	Non-pain GI symptoms	Sweating abnormalities or heat/exercise intolerance	Febrile crises	Patient-reported progression of symptoms/signs
PREDICT-FD^a^ [11]	+	+	+	+	+	+
EFWG^b^ [1]	Diagnostic sign	–	Aged < 16 years All, IIA Aged ≥ 16 years All, IIB			
Australia [24]	–	–	–	–	–	–
Canada [21]	Diagnostic sign	Diagnostic/prognostic	All^c^	–	–	–
Catalonia (Spain)	–	–	–	–	–	–
France^d^ [23, 25]	Diagnostic sign	–	–	–	–	–
Portugal [22]	–	–	All, recurrent diarrhea attributable to FD	All	–	–
Slovenia (FCGHSG)	Diagnostic sign	Confirmatory skin biopsy if needed in asymptomatic boys with classical disease	Postprandial diarrhea^e^	All^e^	–	–
Switzerland^f^	–	–	F	–	–	–
UK^g^ [26]	–	–	–	–	–	–

Unpublished guidelines are summarized in Additional file 1: Table S1

^a^Consensus was reached that FD-specific treatment should be initiated at diagnosis in male patients aged 16 years or older who are asymptomatic for organ involvement, in boys younger than 16 years old with early indicators of organ involvement, and in all patients with guideline indicators of organ involvement

^b^Recommendations are based on class of evidence assigned: class I, treatment recommended or indicated; class IIA, treatment should be considered; class IIB, treatment may be considered; class III, treatment not recommended

^c^Significant GI symptoms unresponsive to other measures for at least 6 months or associated with poor growth or significant reduction in quality of life

^d^All male patients with a confirmed FD diagnosis should be offered ERT from age 18 years

^e^Central and/or autonomic nervous system involvement consistent with FD

^f^ERT is practically always indicated in men, even those with mild symptoms and low organ involvement, and in patients undergoing hemodialysis or with a kidney transplant

^g^FD-specific therapy should be considered in male patients with classical mutations at diagnosis; tabulated additional considerations apply to male and female patients with later-onset disease

+ , achieved consensus in PREDICT-FD; EFWG, European Fabry Working Group; ERT, enzyme replacement therapy; F, female patient(s); FCGHSG, Fabry Center, General Hospital Slovenj Gradec; FD, Fabry disease; GI, gastrointestinal; M, male patient(s)

#### Renal involvement

Indicators of renal damage identified in PREDICT-FD (Table 1) were largely represented in treatment guidelines. Elevated urinary albumin–creatinine ratio and early stages of chronic kidney disease were parameters widely used to inform treatment decisions, even if thresholds for urine protein level and for glomerular filtration rate differed. Histological damage was not part of consensus guidance from the EFWG and, in most countries, biopsy to detect histological damage was noted only as a confirmatory option [1]. Being a sign associated with later-stage disease, proteinuria did not feature in the PREDICT-FD consensus but was nominated by most guidelines.

#### Cardiac involvement

Biomarkers indicative of cardiac damage identified in PREDICT-FD (Table 2) only featured in the guidelines from Canada [21]. Coverage of cardiac signs identifiable with cardiac magnetic resonance imaging (cMRI) varied considerably by country and was absent from the EFWG guidance [1]. Diastolic dysfunction, identified as an early sign of cardiac damage in PREDICT-FD [11], featured in guidance from Canada, Catalonia (Spain), Slovenia, and Switzerland; only Canadian guidance noted short PR interval on electrocardiogram as an early sign [21]. Guidance with respect to cardiac indicators was often generic; only Canadian guidelines provided very specific cardiac damage criteria [21].

#### Neurological involvement

White matter lesions featured in the EFWG guidance [1] and in Portuguese guidelines [22] in female and late-onset male patients; Canadian guidelines noted that the significance of such imaging abnormalities in FD was unclear (Table 3) [21]. Sudden hearing loss, excluded from the PREDICT-FD consensus, featured as possible justification for disease-specific therapy initiation in Canadian, French, Portuguese, and the EFWG guidance [1, 21–23]. Only Canadian guidance noted the potential significance of acute optic neuropathy [21]. Neuropathic pain is widely recognized as an important early sign in FD, although not necessarily as justification for disease-specific therapy initiation. Painful gastrointestinal neuropathy symptoms were noted only in PREDICT-FD [21], although gastrointestinal symptoms (pain, diarrhea) were represented in guidance from Canada, Portugal, Slovenia, and Switzerland.

#### Patient-reported and other signs

Angiokeratoma featured only in guidelines as a supportive diagnostic sign, and only the Canadian and Slovenian guidance noted that biopsies other than renal or cardiac might be helpful aids to diagnosis or to ratify treatment initiation (Table 4) [21]. Only guidance from Portugal included abnormal hidrosis and exercise intolerance as supportive signs for treatment, and no other patient-reported signs (febrile crises, progression of signs/symptoms) featured in any guidelines.

### Diagnosis of FD and treatment initiation

The focus of PREDICT-FD was on indicators for FD-specific treatment initiation in patients who had already received a diagnosis of FD; however, the need for diagnosis (variously based on combinations of biochemical, genetic, and clinical features) is stipulated before treatment in all guidelines reviewed (Additional file 1: Table S1).

### Consensus on factors influencing treatment initiation in PREDICT-FD

The panel rated four statements describing drivers of treatment initiation as ‘important’ and reached consensus on three. Two statements regarding barriers to treatment were deemed important and consensus was reached on both (Table 5).

**Table 5:** Drivers and barriers associated with early treatment initiation in FD that achieved consensus in PREDICT-FD

**Drivers**	
**1. Male sex, young age, and clinical findings, such as severe pain and signs/symptoms of organ involvement**, are key drivers of early initiation of treatment (4.8)	
**2. Improving clinical outcomes and preventing disease progression** are key drivers of early initiation of FD-specific treatment (4.6)	
**3. A family history of FD, especially if severe or with major organ involvement or premature death**, is a key driver of early initiation of treatment (4.4)	

**Barriers**	
**1. A lack of biomarkers predicting which patients will progress and which will respond to treatment** is a key barrier to early initiation of treatment (4.1)	
**2. Misdiagnosis** is a key barrier to early initiation of treatment (3.9)	

Data in parentheses are the mean of agreement scores awarded by 21 panel members based on a 5-point pivoted Likert scale (1 = strongly disagree; 3 = neither agree nor disagree; 5 = strongly agree). The criterion for consensus was an agreement rating of at least 4 awarded by more than 67% of those who voted

Bold indicates the consensus statements describing the possible impact of PREDICT-FD

FD, Fabry disease; PREDICT-FD, PRoposing Early Disease Indicators for Clinical Tracking in Fabry Disease

#### Drivers

Statement 1. “Male sex, young age, and clinical findings, such as severe pain and signs/symptoms of organ involvement, are key drivers of early initiation of treatment.”

Generally, guidelines support the finding in PREDICT-FD that male sex, pain, and organ involvement are drivers of treatment initiation, but age mainly pertains to men with classical disease. All men with classical disease should be offered disease-specific therapy. In several countries, younger asymptomatic male patients with a classical FD diagnosis can be considered for disease-specific therapy: Catalonia (patients aged 16 years or older with classical FD); Portugal and Slovenia (case-by-case basis in asymptomatic male patients with classical FD older than 8 years old); Switzerland (all male patients with pathogenic mutations); and the UK (male patients with classical mutations/phenotype at diagnosis). Other countries recommend considering disease-specific therapy only when symptoms appear: Canada (the presence of a subset of specific renal, cardiac, and neurological signs, as well as intractable gastrointestinal symptoms or neuropathic pain) [21]; France (treatment of asymptomatic boys not justified; treatment considered with early organ involvement or significant pain); and Italy (male patients with classical disease treated at the first sign of organ involvement; treatment considered in asymptomatic boys younger than 16 years old). Australian guidelines do not differentiate between male patients with classical FD and patients with other types of disease for subsidized treatment initiation, but instead stipulate the symptomatology that justifies ERT initiation in male and female patients generally [24].

For other patient groups, Portugal also advocates treatment of any pediatric patient (i.e., not only male patients with classical FD) who presents with certain signs (e.g., renal, cardiac, pain, abnormal hidrosis). Guidance from Catalonia (Spain), Portugal, Slovenia, Switzerland, and the UK associates additional criteria with initiation in women and men with late-onset disease (Additional file 1: Table S1) [26]. These include either evidence of renal, cardiac, or cerebrovascular (except in the UK) involvement for which causes other than FD have been excluded or evidence of specific FD-related complications, such as uncontrolled pain or gastrointestinal symptoms. In the Lazio region of Italy, guidance for female patients with classical disease and male patients with non-classical disease suggests therapy must be started at early clinical signs of organ involvement. Treatment of female patients with non-classical disease may be considered when early FD symptoms appear. In terms of the type of disease-specific therapy recommended, only Australia, Canada, Portugal, and the UK make recommendations about chaperone therapy as well as about ERT. In Canada, ERT rather than chaperone therapy is recommended in children with a confirmed diagnosis because the Canadian regulatory authority has restricted chaperone therapy use to adults (patients aged 16 years or older in the EU and the UK).

Statement 2. “Improving clinical outcomes and preventing disease progression are key drivers of early initiation of FD-specific treatment.”

UK guidance notes that “no trial has yet addressed the appropriate starting time of Fabry-specific therapy or the group of patients most likely to benefit from therapy. However, this is a chronic, slowly progressive disorder and the aim of treatment is to delay/reverse progression or stabilise current parameters. It is anticipated that treatment will be most successful when started early in the course of the disease. Conversely treatment late in the course of the disease may have limited efficacy” [26]. Guidelines from other nations do not typically include statements of this kind, nor do any guidelines mandate optimization of ancillary therapy (e.g., cardioprotective medications or psychosocial support), although some (e.g., Italy) summarize symptomatic therapy options.

Statement 3. “A family history of FD, especially if severe or with major organ involvement or premature death, is a key driver of early initiation of treatment.”

A recommendation to screen for FD among blood relatives of individuals who have received a recent diagnosis does not generally feature in national guidelines. Approval for lysosomal storage disease-specific therapy in Portugal is granted by a national committee, and pedigree analysis of candidates is mandated before they can be proposed. Slovenia and the UK include assessment of family pedigree during follow-up (Additional file 1: Table S1) [26].

#### Barriers

Statement 1. “A lack of biomarkers predicting which patients will progress and which will respond to treatment is a key barrier to early initiation of treatment.”

Plasma levels of Gb3 and LysoGb3 feature in guidelines as diagnostic aids, but guidelines also provide evidence for this being a barrier, in that no biomarkers were available to inform clinicians about when to initiate treatment, likely responsiveness to treatment, or patient prognosis. An association between elevated levels of LysoGb3 and adverse long-term outcomes has been reported and there is evidence that initiating FD-specific treatment earlier rather than later in life is associated with lower LysoGb3 levels [9, 10]. It remains unclear, however, whether treatment-related reductions in LysoGb3 are associated with improved outcomes.

Statement 2. “Misdiagnosis is a key barrier to early initiation of treatment.”

Failure to diagnose FD and misdiagnoses will both delay initiation of treatment, but this problem is more likely caused by a lack of awareness of FD than by shortcomings of treatment guidelines.

### Consensus on the possible impact of PREDICT-FD

Nine statements reached consensus among the PREDICT-FD panel regarding the potential impact of the initiative (Table 6). One of the barriers to treatment (the lack of consolidated biomarkers) featured in two of the statements (1 and 9), timing of therapy was the focus of three statements (2, 3, and 8), and improvements in patient management and, therefore, outcomes were common themes of three more statements (4, 5, and 7).

**Table 6:** Consensus statements describing the possible impact of PREDICT-FD

Impact
1. Findings from the initiative could help to **stimulate research, for example, into predictive biomarkers of disease progression** (4.3)
2. Findings from the initiative could help to **improve communication between HCPs and patients with FD regarding when to start (and stop) disease-specific therapy** (4.1)
3. Findings from the initiative could lead to the **earlier initiation of disease-specific treatment** in patients with FD (4.1)
4. Findings from the initiative could help to **improve outcomes and/or quality of life** of patients with FD (4.0)
5. Findings from the initiative could help to **improve clinical practice and the overall management** of patients with FD (4.0)
6. Findings from the initiative could help to **support negotiations relating to reimbursement of treatment** (4.0)
7. Findings from the initiative could help to **increase HCP awareness and understanding of the need for individualized assessment and regular multi disciplinary follow-up** of patients with FD (4.0)
8. Findings from the initiative could lead to the **achievement of consensus on when to start (and stop) disease-specific treatment** in patients with FD (4.0)
9. Findings from the initiative could lead to **the modification of national treatment guidelines to include predictive biomarkers of disease progression** (3.9)

Data in parentheses are the mean of agreement scores awarded by 21 panel members based on a 5-point pivoted Likert scale (1 = strongly disagree; 3 = neither agree nor disagree; 5 = strongly agree). The criterion for consensus was an agreement rating of at least 4 awarded by more than 67% of those who voted

Bold indicates the consensus statements describing the possible impact of PREDICT-FD

FD, Fabry disease; HCP, healthcare professional; PREDICT-FD, PRoposing Early Disease Indicators for Clinical Tracking in Fabry Disease

### Review of case histories against different guidelines

In total, 17 anonymized case histories from six countries were supplied by panel members, six of whom reviewed some of or all these histories and provided recommendations for treatment initiation based on guidance from their own country, the EFWG, and the PREDICT-FD consensus criteria. The proportions of respondents who recommended treatment in each case are presented in Table 7 and the breakdown by respondent and guideline type is in Additional file 1: Table S3. Panel members were unanimous in supporting the decision to initiate or to continue FD-specific treatment in eight of the 17 cases irrespective of the guidance followed. In only three cases (3, 10, and 12) did the same panel member give different recommendations depending on which guidance was considered. Discrepant recommendations based on different country guidelines were seen in eight cases (1, 3, 5, 8–11, and 15), but discrepant recommendations among panel members were also made based on PREDICT-FD criteria (seven cases: 1, 5, and 8–12) and on the EFWG criteria (seven cases: 1, 3, 5, and 8–11). In most cases, a recommendation to treat was as likely or more likely to be given based on the PREDICT-FD criteria than on the EFWG or country criteria. Case 12 was the only instance of treatment initiation being unanimously recommended based on the EFWG and country guidelines, but not PREDICT-FD criteria.

**Table 7:** Percentage of responding panel members (N = 6) who recommended that treatment should be initiated, based on evaluation of each case history against PREDICT-FD criteria, EFWG guidance, and their own country’s guidelines

Case	PREDICT-FD	EFWG	Country	Case	PREDICT-FD	EFWG	Country
1	3 (60)	2 (50)	3 (60)	10	2 (50)	1 (25)	2 (40)
2	4 (100)	4 (100)	4 (100)	11	2 (67)	2 (67)	3 (75)
3	3 (100)	1 (33)	1 (33)	12	2 (67)	3 (100)	4 (100)
4	3 (100)	3 (100)	3 (100)	13	2 (100)	2 (100)	4 (100)
5	2 (67)	2 (67)	2 (67)	14	3 (100)	3 (100)	4 (100)
6	3 (100)	3 (100)	4 (100)	15	2 (100)	2 (100)	2 (67)
7	2 (100)	2 (100)	3 (100)	16	2 (100)	2 (100)	3 (100)
8	1 (33)	1 (33)	1 (25)	17	2 (100)	2 (100)	3 (100)
9	1 (50)	1 (50)	2 (67)				

Data are n (%). Shaded rows indicate cases for which disease-specific therapy initiation was recommended unanimously, irrespective of which set of guidelines was used

EFWG, European Fabry Working Group; PREDICT-FD, PRoposing Early Disease Indicators for Clinical Tracking in Fabry Disease

## Discussion

Comparison of the PREDICT-FD consensus criteria with wide-ranging international guidance for disease-specific therapy initiation in FD has identified inconsistencies that could be addressed to harmonize early treatment of patients with FD. Differences in indicators of cardiac involvement were particularly evident across the various guidelines reviewed. Delayed treatment initiation because of differences in access to disease-specific therapy may lead to worse outcomes in FD and could be largely avoided if guidelines were aligned. Guidelines revised or issued most recently were most likely to include specific early disease indicators identified in the PREDICT-FD initiative, and only the four most recently issued country guidelines included recommendations about chaperone therapy. Expanding guidance at country level to bring early disease indicators identified in PREDICT-FD to the attention of managing clinicians and updating guideline recommendations to provide, for example, the level of granularity on cardiac indicators seen in Canadian guidance would help to address regional treatment inequalities.

Guidance from Canada and Catalonia (Spain) covered the broadest set of cardiac indicators, and Canadian guidance provided specific criteria for most of these, notably those assessed by cMRI. All the reviewed national or regional guidelines included cardiac indicators, but most could have been defined more precisely or expanded to include other measures. Synthesis of detailed cardiac criteria for treatment initiation in FD may be possible from the various guidelines examined. Countries were mostly aligned on renal indicators, although attitudes to confirmatory renal biopsy varied. The value of such histological evidence is undisputed, but some countries exclude it whereas others recommend it in justifying disease-specific therapy. Most guidelines included metrics for patients with proteinuria, typically a manifestation of FD presenting later than some other renal indicators. However, retaining such indicators in guidance is useful to allow for delayed diagnosis. In terms of neurological signs, few countries considered white matter lesions to be supportive of treatment initiation, Canadian guidelines noting that their clinical significance in FD is unclear.

Among the drivers of treatment initiation identified by PREDICT-FD, some could be more widely addressed in guidelines than currently. Early treatment initiation to optimize clinical outcomes requires early diagnosis of male patients with classical FD and careful follow-up of non-classical male and female patients for early clinical signs/symptoms of organ involvement as described in many guidelines. Studies in untreated symptomatic women with FD indicate that guidelines can be applied inconsistently in different patient groups [29, 30].

The recommendation to treat early is based largely upon expert opinion, so it is important to collect more supportive research data. For example, there may be an optimal age for FD-specific treatment initiation in each patient group. It is likely that techniques such as cMRI and monitoring of biomarkers may not be widely accessible. In these cases, local guidelines could be amended to encourage prompt referral to a regional specialist center. Guidelines could also recommend screening relatives of individuals with a recent diagnosis of FD to try to identify asymptomatic cases requiring follow-up and possibly treatment.

Two barriers to treatment initiation have a substantial impact on effective management of FD: lack of biomarkers and misdiagnosis. Tests for a small number of biomarkers are conducted in some centers but are not widely available, and their prognostic value for disease progression and/or treatment responsiveness remains unclear. LysoGb3 has been associated with negative clinical outcomes in FD [10], and treatment early in life rather than later is associated with lower LysoGb3 levels [9], but direct confirmation of its prognostic value is still needed. Moreover, biomarker tests can yield false-positive results. For example, heterophile antibodies can cause false-positive data in high-sensitivity troponin assays [31], and troponin levels are influenced by factors other than myocardial injury that are common in FD, including cerebrovascular or renal disease [32, 33]. Regarding misdiagnosis, the awareness and index of suspicion of rare diseases are often low among non-specialist clinicians, probably leading to misattribution of FD signs/symptoms and impeding research into prognostic biomarkers.

In addition to considering drivers of and barriers to treatment initiation, the PREDICT-FD expert panel was asked to speculate about the possible impact of the initiative. The need for biomarker research echoed discussions of barriers to treatment and is clearly a source of frustration. However, should PREDICT-FD raise disease awareness, such research endeavors may follow, and findings relating to the timing of treatment initiation and cessation may translate into changes in clinical practice. Early treatment initiation has been the principal focus of PREDICT-FD and expansion of treatment criteria at the national level based on the consensus findings of PREDICT-FD could help to realize this. As well as the potential for improved patient outcomes, harmonizing treatment decisions facilitates consistent communication about such decisions among specialists, patients, and their primary care physicians.

Given the differences among the guidelines reviewed, we undertook a preliminary investigation of how this might affect treatment decisions at the patient level. Expert panel members unanimously supported treatment initiation or continuation in nearly half of the cases reviewed, irrespective of which criteria formed the basis for assessment. Furthermore, in only three cases did applying different guidance cause a panel member to change their decision. Case histories were chosen to exemplify occasions when the decision to treat was equivocal based on country guidance and, in eight cases, different decisions about treatment initiation were reached based on the same criteria. Thus, ambiguity exists in clinical guidelines and emphasizes the utility of a more standardized approach. Increasing the granularity of criteria against which patients are assessed may help to address this. Pragmatism must also be exercised when devising guidance. Anecdotally, there were cases in which confirmatory renal biopsy had been conducted simply to satisfy the requirements of a guideline. This highlights how such invasive procedures can become a barrier to treatment.

Our study has limitations. Comparison of the guideline criteria had to be qualitative because most indicators for treatment initiation were not described or measured uniformly across the guidelines. Comparative performance of case studies against the various guidelines could provide insights about specific differences that translate into treatment delays, but a more structured prospective approach with a larger sample size than used here would be needed. At a superficial level, most of the case histories considered were as likely or more likely to receive a positive treatment recommendation based on PREDICT-FD than on other criteria, but in all cases the sample size was very small. Also, all authors participated in the PREDICT-FD consensus initiative [11], which may have introduced bias to the interpretation of cases. As is common with rare diseases, wider knowledge of FD among general clinicians could accelerate diagnosis, and identification of biomarkers to inform likely outcomes with treatment would improve patient management.

## Conclusions

When an FD diagnosis has been confirmed, disease-specific treatment should be offered as soon as possible to male patients with classical disease, and it should be considered at the earliest signs of organ involvement in other patient groups to try to stabilize or even to reverse disease progression. Although guidance is generally aligned on early indicators of renal involvement, the PREDICT-FD consensus identified several early signs of cardiac involvement not yet widely adopted; Canadian guidelines [21] could be valuable to clinicians seeking cardiac metrics for patient assessment. Comparison of PREDICT-FD and a range of treatment guidelines in FD has revealed considerable scope to harmonize international guidance on treatment initiation. If these findings catalyze changes in clinical practice, they may improve outcomes and quality of life for patients with FD in the long term.

## Methods

Details of the PREDICT-FD Delphi consensus methodology have been reported previously [11]. Briefly, as part of round 1 of the Delphi consensus, the expert panel provided suggestions of key drivers and barriers associated with early initiation of FD-specific treatment and of the possible impact of PREDICT-FD on patients and clinical practice. The panel’s suggestions were compiled by an independent administrator, consolidated into short statements by the non-voting chairs of the initiative, and submitted to the panel for importance rating as part of round 2 of the consensus. Those statements meeting predefined importance criteria in round 2 were resubmitted to the panel for agreement rating as part of round 3; statements meeting agreement criteria in round 3 achieved consensus.

After the PREDICT-FD consensus initiative, panel members were asked to provide local guidelines for disease-specific therapy of FD. National FD treatment guidelines were supplied by panel members from Australia [24], Canada [21], France [23, 25], Portugal [22], and the UK [26]. Unpublished guidelines from Switzerland, for the Lazio region of Italy, and for Catalonia (Spain), and institutional guidelines from the National Fabry Center, General Hospital Slovenj Gradec (Slovenia) were also supplied. The Australian guidelines are for FD-specific treatment subsidized by the national Life-Saving Drugs Program. Guidelines were reviewed and compared to determine areas of discrepancy with PREDICT-FD and to establish what relationship they may have to the identified treatment drivers and barriers. Signs stipulated in guidelines as possible justification for initiating therapy in different patient groups were extracted and compared with those identified in PREDICT-FD [11]. Anonymized patient case histories were provided by the PREDICT-FD co-chairs and by certain panel members after the consensus phase of the initiative was complete. No information regarding patients’ identities was disclosed by the managing physicians to anyone involved in the investigation reported here.

## Supplementary Information

**Additional file 1:** Supplementary data.

## Data Availability

All data generated or analyzed during this study are included in this published article and its Additional files.

## Abbreviations

α-Gal A: Alpha galactosidase A
cMRI: Cardiac magnetic resonance imaging
EFWG: European Fabry Working Group
ERT: Enzyme replacement therapy
EU: European Union
FD: Fabry disease
Gb3: Globotriaosylceramide
*GLA*: Gene encoding α-Gal A
LysoGb3: Globotriaosylsphingosine
PREDICT-FD: PRoposing Early Disease Indicators for Clinical Tracking in FD
