# Supplementary material for: Do clinical guidelines facilitate or impede drivers of treatment in Fabry disease?
Source: Orphanet J Rare Dis. 2022 Feb 8;17:42. doi: 10.1186/s13023-022-02181-4 (PMC8822651; doi:10.1186/s13023-022-02181-4)
Supplement: Supplementary file 1 — Additional file 1: Supplementary data. [file 13023_2022_2181_MOESM1_ESM.docx]

# Supplementary data

**Do clinical guidelines facilitate or impede drivers of treatment in Fabry disease?**

Derralynn A Hughes, Patrício Aguiar, Olivier Lidove, Kathy Nicholls, Albina Nowak,
Mark Thomas, Roser Torra Balcells, Bojan Vujkovac, Michael L West, Sandro Feriozzi

**Table S1** Unpublished treatment initiation guidelines in FD. Published guidelines included in this study were from Australia [1], Canada [2], France [3,4], Portugal [5], and the UK [6].

| **Catalonia (Spain)** | *Initiation owing to disease attributable to FD:*  1. Renal: (a) microalbuminuria; (b) proteinuria >300 mg/24h (children >5 mg/kg/24 h); (c) reduction of GFR in ≥3 determinations to <90 mL/min/1.73m^2^ (calculated using the CKD-EPI equation); (d) patients on renal dialysis and with renal transplant.  2. Cardiac: (a) electrocardiographic alterations: LVH, arrhythmia; (b) echocardiographic alterations: increased LVM, systolic or diastolic dysfunction, echocardiogram with doppler tissue altered in a persistent manner; (c) alteration in cMRI suggestive of deposit.  3. Cerebrovascular: (a) vascular ischemia lesions observed with imaging techniques and not attributable to other causes or risk factors other than FD.  4. Painful neuropathy: (a) chronic pain, uncontrolled with drugs, that alters patient QoL. In patients for whom pain is the only FD symptom, neuropathy must be determined objectively by specific diagnostic tests to initiate substitute enzymatic treatment: electromyogram; evoked potentials; small fibre study; thermotest; microneurography; or nerve biopsy.  5. Men aged >16 years with classical FD but not presenting any clinical complications described above will be considered for treatment. Classical FD is defined as: a definitively pathogenic mutation to the *GLA* gene; absence or minimal activity of alpha-galactosidase; and presence of ≥1 of: angiokeratomas; verticillate cornea; very high Gb3/LysoGb3 levels; biopsy  *Discontinuation will be considered in patients:*   - with no improvement in, or stabilization of, the signs/symptoms that prompted treatment initiation - who only present advanced cardiac involvement with extensive myocardial fibrosis - presenting terminal renal insufficiency, with no renal transplant option, and advanced heart failure (NYHA Class IV) - with cognitive deterioration or life expectancy for which the administration of ERT has no demonstrated benefit for QoL or survival - with no response during 1 year of treatment when the only indication has been neuropathic pain and while also receiving best supportive treatment (exception: men with classical FD) - experiencing serious life-threatening complications relating to administration of agalsidase alpha or agalsidase beta - presenting with a new illness that compromises treatment response with inadequate treatment adherence or inappropriate adherence to monitoring protocols. | |
| --- | --- | --- |
| **Lazio (Italy)**  (translated extract) | Diagnostic suspicion requires integration of a detailed personal and family history, an accurate physical examination and a careful evaluation of blood chemistry tests.  Enzyme activity of α-galactosidase: in the presence of clinical suspicion, it is necessary to determine enzymatic activity in leukocytes from peripheral venous blood, or on dried blood spots, or in fibroblasts. Laboratory diagnosis is confirmed by demonstration of a marked enzyme deficiency in hemizygous males. Enzyme level in females can sometimes facilitate identification of heterozygotes but is often unreliable as a diagnostic test due to a mosaic effect due to the random inactivation of the X chromosome in some tissues and not in others. For the purposes of diagnosis, therefore, it is necessary to genetically characterize the patient.  Molecular analysis of mutations: after finding reduced enzymatic activity, it is essential to proceed with molecular analysis of mutations of the *GLA* gene, for a better definition of the disease. Diagnosis of FD can be difficult in subjects with *GLA* mutations in the absence of phenotypic or biochemical characteristics of the disease. A complex and careful clinical-biochemical-genetic evaluation by specialists experienced in FD is therefore necessary.  FD therapy is based on ERT with recombinant alpha galactosidase. Two formulations are currently on the market: agalsidase beta and agalsidase alfa. Both have been approved by the European Agency for the Evaluation of Medical Products since 2001 while the only agalsidase beta is currently approved by the Food and Drug Administration in the USA.  The agalsidase alfa enzyme is infused at a dose of 0.2 mg / kg in 40 minutes every 15 days while agalsidase beta is administered at a dose of 1 mg / kg every 14 days. There are no studies that have really shown the superiority of one enzyme over the other. In general, TES can stop or attenuate the progression of the disease.  The criteria for starting or stopping ERT have recently been discussed and shared by a group of experts. For affected males in classical form, the consensus reached was that ERT is recommended as soon as there are the first clinical signs of heart, kidney or brain involvement, but it can be considered in patients over 16 years of age in the absence of clinical signs or symptoms of organ involvement. Classically affected females and males with non-classical Fabry disease should be treated as soon as there are the first clinical signs of heart, kidney or brain involvement, while treatment can be considered in females where early attributable clinical signs emerge at Fabry's disease.  Of course, these are general recommendations that can be used as a reference for initiation of treatment, but the therapy must be individualized in accordance with the patient's clinical conditions. A careful evaluation of possible initial signs of organ involvement must guide the therapeutic choice in females.  ERT can be administered at the local health company or at reference centres. There is also the possibility, after evaluation by the specialist, that the patient, after having performed a minimum of 10 infusions in the hospital without complications, can take advantage of the home therapy service provided for all patients in the Lazio Region.  Symptomatic therapies for FD include:  - neuropathic pain medications (phenytoin, carbamazepine; gabapentin)  - proteinuria drugs (ACE inhibitors)  - low sodium and protein diet and antihypertensives in case of high blood pressure  - kidney transplant in kidney failure  - platelet anti-aggregants or oral anticoagulants in patients at risk of ischemic events;  - low fat diet and pancreatic enzymes in people with malabsorption  Being a multisystem disease, periodic evaluation of patients with FD must include periodic, clinical and instrumental monitoring of the various organs and systems involved. The timing of these tests will vary according to age, the clinical picture at onset and the expected phenotype in the family.  Close monitoring of cardiac and renal function is essential. In children in whom monitoring through 24-hour urinary collection is not easily achievable, it is possible to use as an average three determinations of microalbumin over 3 urinations. | |
| **Slovenia** | *Diagnosis and screening*  In men, diagnosis is gradual. First, the enzymatic activity of α-Gal A in leukocytes or from a DBS on filter paper is determined. In the case of low or decreased enzyme activity, molecular gene analysis of the *GLA* gene is also performed. In women, determination of enzyme activity is unreliable for the recognition of FD, so molecular genetic analysis of the *GLA* gene is used as the first-choice test in women with suspected FD.  We recommend determining the enzyme activity of α-Gal A in leukocytes or with use of DBS. In the case of the latter, an additional confirmatory analysis of α-Gal A enzyme activity in a leukocyte sample is recommended to rule out possible false-positive results. Simultaneously with the analysis of α-Gal A enzyme activity, an additional lysosomal enzyme (e.g. β-glucosidase) should be analysed in each sample to rule out possible false-negative results.  In the case of an identified mutation, the type of mutation and its pathogenicity are defined. Prior to performing molecular genetic testing, the subject or his/her guardian in the case of minors, must sign an informed consent to the analysis. As part of the molecular genetic analysis of the *GLA* gene, it is necessary to determine the nucleotide sequence of the entire coding region of the gene together with the boundaries of exons with introns (e.g. by Sanger sequencing or next-generation sequencing). Defined genetic mutations are interpreted according to the guidelines of the American Medical Genetics Association, changes are checked in databases of pathological changes, in databases containing data on the variability of the human genome in the seemingly healthy population, and then evaluated (pathological, benign, variant of unknown significance). In the event that the causality of the identified genetic variant is unclear and it is a GVUS, additional clinical investigations, including organ biopsies, will be performed. Excluding other causes represents definitive confirmation of the disease.  A classical mutation is defined as that in which a gene mutation in the *GLA* gene (both sexes) is confirmed.  In men, enzyme activity <5% with at least one FD sign present:   - angiokeratoma - cornea verticillata - acroparesthesias - elevated serum and/or urine LysoGb3 or Gb3 (values are in the range of known values for men with “classic” FD) - a relative with pre-defined classic disease according to the above criteria.   In women, at least one FD sign:   - angiokeratoma - cornea verticillate - acroparesthesia - elevated serum and/or urine LysoGb3 or Gb3 (values are in the range of known values for women with “classic” FD) - relative with pre-defined classic disease according to the above criteria.   All patients with established FD should be offered genetic counselling, which can be performed by a physician with appropriate knowledge of genetics or a clinical geneticist. A consultation with a clinical geneticist is advised before a planned pregnancy or in case of family planning. In patients with confirmed FD, accurate family history data should be collected and arranged in the form of a family tree (pedigree), which is the basis for further cascade screening of family members, which also includes testing of asymptomatic family members. If we know which mutation in the family causes the disease, it makes sense to immediately genetically test potential patients. Testing of asymptomatic children is performed in agreement and with the consent of the parents. It is recommended that testing be done before the age of 8 for boys and 16 for girls. Neonatal screening for FD as part of routine neonatal screening is not recommended in accordance with current disease knowledge.  We recommend testing groups “at risk” for FD. Patients with:   - nephropathy of unknown cause - hypertrophic cardiomyopathy of unknown cause - cryptogenic stroke - multiple sclerosis (men + other signs of FD: acroparesthesias, angiokeratomas, hearing loss, anhidrosis, cornea verticillata) - signs characteristic of FD: angiokeratomas or cornea verticillate - at least 3 other accompanying signs of FD: chronic gastrointestinal problems (diarrhea, abdominal pain or cramps), hypohidrosis, heat intolerance, lymphedema, hearing loss or tinnitus, postural hypotension, unexplained white matter changes on MRI, basilar dolichoectasia, ischemic optic neuropathy, unexplained joint and/or muscle pain in extremities - a history of clinically possible FD in a family where relatives could not be tested.   Screening tests for risk groups are done with use of DBS. Important: observing correct sampling and use of the prescribed filter paper. For men, diagnosis is staged: first we measure the enzymatic activities from the DBS, and if positive, genetic analysis follows. In women, genetic analysis is done immediately. All adult patients with a positive result for FD are referred to the national Fabry Center, General Hospital Slovenj Gradec and all children to the Pediatric Clinic of the University Medical Center Ljubljana for further diagnostic and clinical treatment. Final confirmation of Fabry disease is made by the team of experts at the national Fabry Center, General Hospital Slovenj Gradec and in the case of children together with experts from the Pediatric Clinic of the University Medical Center Ljubljana.  *Disease-specific treatment initiation*  Treatment is initiated when approved by a team of experts (council) at the national Fabry Center, General Hospital Slovenj Gradec. Signatures of at least 4 members are required. For children, at least one member of the council is always an expert pediatrician from the Pediatric Clinic of the University Medical Center Ljubljana.   - The decision on treatment is made unanimously - The choice of disease-specific drug for the treatment of FD is made by the council of the Fabry Center, General Hospital Slovenj Gradec. - Medicines registered in the Republic of Slovenia are used for disease-specific treatment of patients with FD - The use of unregistered drugs for treatment of FD must be approved by the council of the Fabry Center, General Hospital Slovenj Gradec   Criteria for initiating treatment of the classic form of FD  Males with symptoms of FD (any of the following):   - acroparesthesia and pain - postprandial diarrhea and abdominal pain - Heart: hypertrophic cardiomyopathy, diastolic dysfunction, signs of fibrosis - Kidney: chronic kidney disease regardless the stage, albuminuria and/or proteinuria - Neurological signs: central and/or autonomic nervous system involvement consistent with Fabry disease   Women with a classical mutation are monitored. The decision to start disease-specific treatment is issued by the council of the Fabry Centre, General Hospital Slovenj Gradec according to the development (signs of progression) of the disease and the results of the clinical examination (presence of at least 1 sign of FD listed above) and assessments and/or biomarker levels (eg serum LysoGb3). If necessary, a biopsy (kidney, heart) is performed in borderline cases.  Children with a classic mutation are treated at onset of first symptoms (regardless of sex). In asymptomatic boys with a known mutation that causes the classic course of the disease, treatment can also be chosen in the asymptomatic period after 8 years of age. Evidence of significant lysosomal accumulation of globotriaosylceramide in the organs (if necessary, a biopsy [kidney, heart, skin] is performed) and/or elevated serum and/or urine LysoGb3 or Gb3 (values in the range of known values for the “classic” FD) and a positive decision by the council of the Fabry Center, General Hospital Slovenj Gradec are required to initiate treatment.  Criteria for initiating treatment of the late-onset form of FD  Men and women with late mutations are monitored. The decision to initiate treatment is issued by the council of the Fabry Center, General Hospital Slovenj Gradec according to the development (disease progression) of the disease and the results of the clinical examination (presence of at least one of the signs of FD listed above) and assessments and/or biomarker levels (eg LysoGb3 in serum). If necessary, a biopsy (kidney, heart) biopsy is performed in borderline cases.  *Criteria not to initiate disease-specific therapy*   - Advanced heart disease with extensive fibrosis if heart failure is the only indication for therapy - End-stage renal disease without the possibility of transplantation, combined with advanced heart failure NYHA Class IV. - Any disease with a life expectancy <1 year. - Severe cognitive decline of any cause.   *Criteria for discontinuation of ERT*   - at the patient's request - in advanced FD if the prognosis is poor and the expected survival is short. (End-stage renal failure without transplantation option in combination with advanced NYHA Class IV heart failure) - in any disease condition if the prognosis of the disease is considered poor and the expected survival is short (<1 year) - non-participation of the patient (received <75% of infusions in the last year) - present conditions and habits that significantly affect the patient's participation in the treatment process (alcoholism, drug use, etc.) - severe cognitive decline of any cause - permanent migration to another country.   The decision to discontinue enzyme therapy is made in agreement with the patient and their relatives  *Disease non-specific treatment*  Diseases and complications of FD (e.g. heart failure, heart rhythm disorders, chronic kidney disease, proteinuria, stroke) are treated according to the recommendations that apply to these conditions in patients of other aetiologies.  Heart rhythm disorders   - Avoid chronic use of drugs that may affect the metabolism of α-Gal A (e.g. amiodarone, chloroquine). Caution is advised when using beta-blockers, especially in the case of recorded bradycardia - ICD insertion is indicated in patients after surviving VT/VF congestion or with hemodynamically significant VT and expected survival > 1 year (not as primary prevention)   Anticoagulant and antiaggregation therapy   - Anticoagulant therapy is recommended for all HCMP and all forms of AF. It is advisable to consider the introduction of anticoagulant therapy also in patients with AF without myocardial hypertrophy - The choice of anticoagulant or antiplatelet agent applies as to patients without FD. | |
| **Switzerland** | *Male patients*  ERT is practically always indicated, even with mild symptoms and low organ involvement. To avoid further complications (e.g., stroke, systolic/diastolic heart failure), ERT is also indicated in patients on hemodialysis or after kidney transplantation | *Female patients*  The time to start ERT should be individual for heterozygous women. ERT should be recommended for: (a) therapy-resistant pain; (b) manifest diastolic dysfunction, LVH, arrhythmias, which can be attributed to cardiac involvement  (c) proteinuria > 300 mg/24h, regardless of kidney disease stage; (d) cerebrovascular manifestations (insult, TIA), dizziness; (e) gastrointestinal complaints (abdominal pains or cramping, diarrhea); (f) relevant, histologically proven Gb3 deposits in kidney or heart biopsies |

α-Gal A, α-galactosidase A; ACE, angiotensin-converting enzyme; AF, atrial fibrillation; cMRI, cardiac magnetic resonance imaging; CKD-EPI, Chronic Kidney Disease Epidemiology Collaboration; DBS, dry blood spot; ERT, enzyme replacement therapy; FD, Fabry disease; Gb3, globotriaosylceramide; GFR, glomerular filtration rate; *GLA*, α-galactosidase A gene; LV, left ventricular; GVUS, genetic variant of unknown significance; HCMP, hypertrophic cardiomyopathy; ICD, implantable cardioverter defibrillator; LVH, LV hypertrophy; LVM, LV mass; LysoGb3, globotriaosylsphingosine; NYHA, New York Heart Association; QoL, quality of life; TIA, transient ischemic attack; VF, ventricular fibrillation; VT, ventricular tachycardia

**Table S2** Guidelines for treatment eligibility by organ involvement

| **Renal disease** | | |
| --- | --- | --- |
| **Australia** [1] | Confirmation by biopsy is recommended for all patients to provide prognostic information, exclude other causes of nephropathy, demonstrate evidence of focal glomerular sclerosis or fibrosis greater than that expected for age, once other causes of nephropathy have been excluded, and to document significant histological changes related to FD | |
|  | *Male patients*   - abnormal albuminuria (> 20 µg/min) determined by two separate samples ≥24 h apart, and/or - abnormal protein excretion (>150 mg/24 h), and/or - urinary albumin:creatinine ratio >ULN in two separate samples, ≥24 h apart, and/or - renal disease due to long-term accumulation of glycosphingolipids in the kidneys. | *Female patients*   - proteinuria >300 mg/24 h with clinical evidence of progression - renal disease due to long-term accumulation of glycosphingolipids in the kidneys. |
| **Canada** [2] | One major criterion required…  Fabry nephropathy with reduced GFR   - For GFR < 60 ml/min/1.73m^2^ CKD stages 3-5: at least 2 consistent estimates or measurements of GFR over a minimum of 2 months. - For GFR 60 - 90 ml/min/1.73m^2^, CKD stage 2: at least 3 consistent estimates or measurements of GFR over at least 4 months with a GFR slope greater than age-related normal. - For GFR >135 ml/min/1.73m^2^: a 15% decrease in GFR or a GFR slope greater than age-related normal as measured by nuclear medicine technique. Estimated GFR is not accurate in this range and thus cannot be used. - Persisting proteinuria of 500 mg/day/1.73m^2^ or greater without other cause. - Findings of high-risk pathology (glomerular sclerosis, tubulointerstitial atrophy, fibrosis or vascular sclerosis) on renal biopsy are a major criterion in males only (see comments). | …or two minor criteria required  Hyperfiltration   - There should be at least two consistent measurements of GFR by nuclear medicine techniques at least one month apart when GFR reaches or exceeds 135 ml/min/1.73m^2^. Hyperfiltration by eGFR as calculated by any formula is not accurate and thus not acceptable.   Isolated proteinuria   - Isolated proteinuria of 300 mg/day/1.73m^2^ or greater than normal for age and gender and persistent for at least one year with exclusion of other causes.   Renal tubular dysfunction.   - Fanconi syndrome and/or nephrogenic diabetes insipidus confirmed usually with abnormal water deprivation test and resistance to DDAVP.   Hypertension of at least 1-year duration.  Renal pathology   - This may be taken into account in women as a minor criterion if the patient has indications for renal biopsy. If a renal biopsy is done, the presence of glomerular sclerosis, tubulointerstitial atrophy and fibrosis or vascular sclerosis should be considered a minor criterion in women |
| **Catalonia (Spain)** | (a) microalbuminuria  (b) proteinuria >300 mg/24h (children >5 mg/kg/24 h)  (c) reduction of GFR in ≥3 determinations to <90 mL/min/1.73m^2^ (calculated using the CKD-EPI equation)  (d) patients on renal dialysis or with renal transplant | |
| **France** [3,4] | – | |
| **Lazio (Italy)** | Serum creatinine, serum electrolytes, BUN, urinary protein/creatine ratio, microalbuminuria, urinary Gb3 (optional)  CKD stage 1 or 2 with proteinuria >1 g/d or stage IV check every 3 months  CKD stage 1 or 2 with proteinuria <1 g/d check every 12 months  CKD stage 3 check every 6 months | |
| **Portugal** [5] | Paediatric, male late-onset and female classical or late-onset  (a) microalbuminuria or proteinuria  (b) GFR <90 mL/min/1.73m^2^  Adult, male, late-onset and female classical or late-onset  (a) microalbuminuria or proteinuria attributable to FD (may require kidney biopsy)  (b) GFR <90 mL/min/1.73m^2^ | |
| **Slovenia**  **(FCGHSG)** | CKD (regardless of stage) albuminuria and/or proteinuria | |
| **Switzerland** | Female patients with proteinuria > 300 mg/24 h, regardless of kidney disease stage | |
| **UK** [6] | (a) CKD stage 3 based on ≥2 consistent estimates or measured GFR over a minimum of 6 months  (b) CKD stage 2 based on ≥3 consistent estimates or measured GFR over at least 12 months with a GFR slope greater than age-related normal  (c) persistent proteinuria >300 mg/24 h for males. Females seldom progress to end-stage renal disease; if proteinuria is the only presentation, anti-proteinuria medications (ACE/ARB) should be tried in the first instance for a minimum of 12 months. | |
| **Cardiac disease** | | |
| **Australia** [1] | Confirmation by myocardial biopsy is recommended to exclude other causes of cardiac hypertrophy  (a) LVH, as evidenced by cMRI or echocardiogram data, in the absence of hypertension. If hypertension is present, it should be treated optimally for ≥6 months prior to the submission of an application through this criterion  (b) significant life-threatening arrhythmia or conduction defect | |
| **Canada** [2] | Two criteria required   - LV wall thickness >12 mm in males and >11 mm in females - LV hypertrophy by Estes ECG score must be greater than 5 - LV mass index by 2D echo 20% above normal for age - Increase of LV mass of at least 5 g/m2/year, with three measurements over a minimum of 12 months - Diastolic filling abnormalities by 2D echocardiogram, Grade 2 or Grade 3 diastolic dysfunction as outlined by ASE and/or the presence of speckle tracking abnormalities - Abnormal base to apex circumferential strain gradient - Increased LA size on 2D echo. In parasternal long axis view (PLAX) >40 mm; Left atrial volume index > 34 mL/m^2^ - Cardiac conduction and rhythm abnormalities: AV block, short PR interval, left bundle branch block, ventricular or atrial tachyarrhythmias, sinus bradycardia (in the absence of drugs with negative chronotropic activity or other causes) - Moderate to severe mitral or aortic insufficiency - Late enhancement of left ventricular wall on cardiac MRI - T1 values using a 1.5 Tesla magnet in males below 901 ms and females below 916 ms - Increase of either NT-proBNP above the upper limit of normal for age and gender OR an increase of high sensitivity troponin (a surrogate marker of fibrosis) more than 2 times the upper limit of the normal range | |
| **Catalonia (Spain)** | (a) electrocardiographic alterations: LVH, arrhythmia  (b) echocardiographic alterations: increased LVM, systolic or diastolic dysfunction, echocardiogram with doppler tissue altered in a persistent manner  (c) alteration in cMRI suggestive of deposit. | |
| **France** [3,4] | – | |
| **Lazio (Italy)** | ECG, Echocardiogram 2D with doppler  ECG Holter if palpitations or arrhythmia  cMRI (adults)  Coronary angiography (children) | |
| **Portugal** [5] | Paediatric, male late-onset and female classical or late-onset  (a) cardiomyopathy  (b) arrhythmia  Adult, male late-onset and female classical or late-onset  (a) symptomatic cardiac disease (dyspnoea, palpitations syncope, thoracic pain)  (b) left ventricular hypertrophy  (c) cardiac rhythm or conduction disturbance  (d) myocardial fibrosis attributable to FD | |
| **Slovenia (FCGHSG)** | hypertrophic cardiomyopathy, diastolic dysfunction, signs of fibrosis | |
| **Switzerland** | Female patients with manifest diastolic dysfunction, LVH, or arrhythmias which can be attributed to cardiac involvement | |
| **UK** [6] | (a) LV wall thickness >13 mm in males and >12 mm in females  (b) LV mass index by 2D echo/cMRI above normal for age and sex  (c) late gadolinium enhancement on cMRI. | |
| **Cerebrovascular disease** | | |
| **Australia** [1] | Ischemic vascular disease shown on objective testing with no other cause or risk factors identified | |
| **Canada** [2] | (see FD complications) | |
| **Catalonia (Spain)** | Vascular ischemia lesions observed with imaging techniques and not attributable to other causes or risk factors other than FD | |
| **France** [3,4] |  | |
| **Lazio (Italy)** | (see FD complications) | |
| **Portugal** [5] | Adult, male, late-onset and female classical or late-onset  (a) stroke or TIA  (b) silent cerebral infarction | |
| **Slovenia (FCGHSG)** | – | |
| **Switzerland** | Female patients with cerebrovascular manifestations, such as insult or TIA, or dizziness | |
| **UK** [6] | – | |
| **FD complications** | | |
| **Australia** [1] | Uncontrolled chronic pain despite use of maximum doses of appropriate analgesia and antiepileptic medications for peripheral neuropathy. Patients meeting this criterion must provide ongoing evidence of effect, through analgesic intake, pain diary, summary letter from treating physician | |
| **Canada** [2] | One neurological disease criterion required   - stroke or TIA documented by a neurologist diagnosed on the basis of clinical features (TIA) and/or CNS imaging criteria consistent with the diagnosis of stroke - sudden onset unilateral hearing loss when other possible causes have been excluded - acute ischemic optic neuropathy when all other possible causes have been excluded.   Neuropathic pain is not an indication for disease specific therapy in isolation, but a 1-year trial could be given if certain outcome criteria are stipulated by the clinician, and agreed by the patient, that must be met for treatment to continue beyond 1 year.  Significant gastrointestinal symptoms unresponsive to other measures for at least 6 months or associated with poor growth or significant reduction in quality of life. | |
| **Catalonia (Spain)** | Painful neuropathy: chronic pain, uncontrolled with drugs, that alters patient QoL. In patients for whom pain is the only FD symptom, neuropathy must be determined objectively by specific diagnostic tests to initiate substitute enzymatic treatment: electromyogram; evoked potentials; small fibre study; thermotest; microneurography; or nerve biopsy. | |
| **France** [3,4] | – | |
| **Lazio (Italy)** | Clinical assessment for acroparesthesia, weakness, fever, hot/cold intolerance signs and symptoms of TIA or stroke  Brief Pain inventory test  MRI at first visit and when indicating for TIA/stroke; in all females every 3 years  Angio-MRI if thrombotic events are suspected  Check for risk factors (lipid profile, plasma homocysteine) every 1–2 years | |
| **Portugal** [5] | Paediatric, male late-onset and female classical or late-onset  (a) neuropathic pain, pain crisis or FD neuropathy  (b) abdominal pain or recurrent diarrhea  (c) anhidrosis / hypohidrosis and exercise intolerance  Adult, male, late-onset and female classical or late-onset  (a) neuropathic pain, pain crisis or Fabry disease neuropathy  (b) cerebral white matter lesions  (c) hearing loss corrected for age  (d) recurrent diarrhea attributable to FD  (e) anhidrosis / hypohidrosis and exercise intolerance | |
| **Slovenia (FCGHSG)** | acroparesthesias and pain  postprandial diarrhea and abdominal pain  central and/or autonomic nervous system involvement consistent with FD | |
| **Switzerland** | Female patients with  (a) therapy-resistant pain  (b) gastrointestinal complaints (abdominal pains or cramping, diarrhea)  (c) relevant, histologically proven Gb3 deposits in kidney or heart biopsies | |
| **UK** [6] | (a) uncontrolled pain or gastrointestinal symptoms leading to a need to alter lifestyle or that interferes with QoL. Patients whose sole eligibility criterion is pain should have been assessed by a specialist pain team. Patients whose sole eligibility criterion is GI symptoms should have been assessed by a specialist GI team  (b) if FD-related symptoms are the only indication for consideration of FD-specific therapy a trial could be given for a year with prespecified outcomes agreed by the treating physician and the patient as to what would constitute a positive effect for symptom control. Such outcomes may include: (i) reduction in the need for analgesics; (ii) reduction in time lost from work, (iii) significant improvements in validated pain scoring and or QoL measures. | |

ACE/ARB, angiotensin converting enzyme/angiotensin-receptor blockers; BUN, blood-urea nitrogen; CKD, chronic kidney disease; cMRI, cardiac magnetic resonance imaging; ECG, electrocardiogram; eGFR, estimated GFR; FCGHSG, Fabry Center, General Hospital Slovenj Gradec; FD, Fabry disease; Gb3, globotriaosylceramide; GFR, glomerular filtration rate; GI, gastrointestinal; LV, left ventricular; LVH, LV hypertrophy; NT-proBNP, N-terminal pro-natriuretic brain peptide; QoL, quality of life; RFH, Royal Free Hospital; TIA, transient ischemic attack; ULN, upper limit of normal

**Table S3** Case study evaluation by respondent and guideline type

| **Case** | **Respondents, PREDICT-FD guideline** | | | | | | **Respondents, EFWG guideline** | | | | | | **Respondents, country guidelines** | | | | | |
| --- | --- | --- | --- | --- | --- | --- | --- | --- | --- | --- | --- | --- | --- | --- | --- | --- | --- | --- |
|  | 1 | 2 | 3 | 4 | 5 | 6 | 1 | 2 | 3 | 4 | 5 | 6 | 1 | 2 | 3 | 4 | 5 | 6 |
| 1 |  | Y | N | N | Y | Y | Y |  | N | N | Y |  | Y | Y | N | N | Y |  |
| 2 | Y | Y |  | Y |  | Y | Y | Y |  | Y |  | Y | Y | Y |  | Y |  | Y |
| 3 | Y | Y |  | Y |  |  | Y | N |  | N |  |  | N | N |  | Y |  |  |
| 4 | Y | Y |  | Y |  |  | Y | Y |  | Y |  |  | Y | Y |  | Y |  |  |
| 5 |  | N |  | Y |  | Y |  | N |  | Y |  | Y |  | N |  | Y |  | Y |
| 6 |  | Y | Y | Y |  |  |  | Y | Y | Y |  |  | Y | Y | Y | Y |  |  |
| 7 |  | Y |  | Y |  |  |  | Y |  | Y |  |  | Y | Y |  | Y |  |  |
| 8 |  | N | Y | N |  |  |  | N | Y | N |  |  | N | N | Y | N |  |  |
| 9 |  | N |  | Y |  |  |  | N |  | Y |  |  | Y | N |  | Y |  |  |
| 10 |  | N | N | Y |  | Y |  | N | N | Y |  | N | Y | N | N | Y |  | N |
| 11 |  | N |  | Y |  | Y |  | N |  | Y |  | Y | Y | N |  | Y |  | Y |
| 12 |  | N |  | Y | Y |  |  | Y |  | Y | Y |  | Y | Y |  | Y | Y |  |
| 13 |  | Y |  | Y |  |  |  | Y |  | Y |  |  | Y | Y |  | Y | Y |  |
| 14 |  | Y | Y | Y |  |  |  | Y | Y | Y |  |  | Y | Y | Y | Y |  |  |
| 15 |  | Y |  | Y |  |  |  | Y |  | Y |  |  | N | Y |  | Y |  |  |
| 16 |  | Y |  | Y |  |  |  | Y |  | Y |  |  | Y | Y |  | Y |  |  |
| 17 |  | Y |  | Y |  |  |  | Y |  | Y |  |  | Y | Y |  | Y |  |  |

Shaded case rows indicate unanimity among the respondents, irrespective of the guideline chosen.

EFWG, European Fabry Working Group; N, do not treat; PREDICT-FD, PRoposing Early Disease Indicators for Clinical Tracking in Fabry Disease; Y, treat.

**References**

1. Life Saving Drugs Program – Fabry disease – Guidelines – October 7, 2020. Available at: <https://www.health.gov.au/sites/default/files/documents/2020/11/life-saving-drugs-program-fabry-disease-guidelines.pdf>. Accessed, August 25, 2021.
2. Canadian Fabry Disease Treatment Guidelines. Available at: <https://garrod.ca/wp-content/uploads/2020/02/Canadian-Fabry-Treatment-Guidelines-2019-final.pdf>. Accessed August 25, 2021.
3. Lidove O, Bekri S, Goizet C, Khau Van Kien A, Aractingi S, Knebelmann B et al. [Fabry disease: proposed guidelines from a French expert group for its diagnosis, treatment and follow-up]. Presse Med. 2007;36(7-8):1084–97.
4. Maladie de Fabry – Protocole national de diagnostic et de soins [Fabry disease – National Diagnostic and Care Protocol]. Available at: <https://www.has-sante.fr/upload/docs/application/pdf/2010-12/ald_17_pnds_fabry_vd.pdf>. Accessed August 25, 2021.
5. [Specific therapy for Fabry disease]. Available at: <http://www.insa.min-saude.pt/wp-content/uploads/2019/06/TerapeuticaDoencaFabry.pdf>. Accessed August 25, 2021.
6. Guidelines for the treatment of Fabry Disease. Available at: <https://bimdg.org.uk/store/lsd/FabryGuide_LSDSS_Jan2020_700523_11032020.pdf>. Accessed August 25, 2021.
